# Supplementary material for: Predictors of hospital expenses and hospital stay among patients undergoing total laryngectomy: Cost effectiveness analysis
Source: PLoS One. 2020 Jul 16;15(7):e0236122. doi: 10.1371/journal.pone.0236122 (PMC7365401; doi:10.1371/journal.pone.0236122)
Supplement: Attachment — Submitted filename: PLOS ONE - June 13 2020.docx [file pone.0236122.s002.docx]

The authors investigated the predictive factors of PO hospital stay & medical cost in patients who benefited from TL. They identified several factors associated with the PO hospital stay or medical cost, including postoperative Hb level, albumin, preoperative RT (salvage TL), cirrhosis history or wound closure method. The study is well-conducted and designed and helpful for OTOHNS surgeons. The paper is short and to-the-point, which is particularly important for a potential publication in the journal.

Some point has however been addressed to improve the manuscript.

-Abstratc: develop the method section: state the studied outcomes (preop RT, etc.).

-Introduction: to the point, well organized.

-Methods: what were your indication to make a flap and which flap were used ? Specify the exclusion criteria.

Line 80: you already define BMI in the abbreviation (line 70), just keep in this line BMI.

-Results: line 114 = the cost is presented in Taiwan money and U.S. dollar. Please, present in the brackets the Euro value for European Physicians. Thus, you cover both U.S. and Europe.

Briefly specify the RT protocol (number of Gy, fraction, etc.)

-Discussion:

Another biological point that is important to consider is the thyroid status. In practice, we usually observed that hypothyroidism patients have wound healing disorder and longer hospital stay. The lack of consideration of this point is a limitation that has to be addressed.

Develop the limitation paragraph, heterogeneity between patients (indication/tumor localization, RT) etc. that may consist of heterogeneity in the study. The inclusion of non-SCC (papillary thyroid) is another bias.

-Conclusion: remove “in total”. The sentence could be: Previous RT history and postoperative lower albumin level significantly impact the  length of hospital stay.
