# Supplementary material for: Predictors of hospital expenses and hospital stay among patients undergoing total laryngectomy: Cost effectiveness analysis
Source: PLoS One. 2020 Jul 16;15(7):e0236122. doi: 10.1371/journal.pone.0236122 (PMC7365401; doi:10.1371/journal.pone.0236122)
Supplement: Attachment — Submitted filename: Response to Reviewers.docx [file pone.0236122.s004.docx]

**Wednesday, June 24, 2020**

**Giovanni Cammaroto**

**Academic Editor, PLOS ONE**

Dear Dr. Cammaroto and colleagues,

We would like to sincerely thank you and the rest of your team for carefully reviewing our manuscript. On the following pages, you will find our point-by-point responses to the reviewers. We believe that our manuscript has been made much stronger, and hope that it is acceptable for publication in your esteemed journal.

We look forward to your kind response.

Sincerely yours,

Chih-Yen Chien, MD, FACS

Professor, Department of Otolaryngology

Chang Gung Memorial Hospital-Kaohsiung Medical Center

123, Ta-Pei Road, Niao-Song District

Kaohsiung, 833

Taiwan

Tel: +886-7-7317123 ext. 2533

Fax: +886-7-7313855

E-mail address: cychien3965@adm.cgmh.org.tw

**Reply to reviewers’ comments**

**To Reviewer #1:**

A well-written article.

Results are very difficult to extrapolate, because as the authors highlight cost related to surgery varies according to each country.

However, add interesting information in order to increase savings related to healthcare.

Response:

We thank the reviewer for this great point. It is true that different health care systems in different countries will affect the length of postoperative hospital stay and inpatient medical expenses after total laryngectomy. We add this in the discussion. (line 241-242)

**To Reviewer #2:**

The authors investigated the predictive factors of PO hospital stay & medical cost in patients who benefited from TL. They identified several factors associated with the PO hospital stay or medical cost, including postoperative Hb level, albumin, preoperative RT (salvage TL), cirrhosis history or wound closure method. The study is well-conducted and designed and helpful for OTOHNS surgeons. The paper is short and to-the-point, which is particularly important for a potential publication in the journal.

Some point has however been addressed to improve the manuscript.

Abstract:

1. Develop the method section: state the studied outcomes (preop RT, etc.).

Response: We thank the reviewer for this suggestion. We added this description about studied outcomes in section of abstract. (line 7 - 12)

Introduction:

2. To the point, well organized.

Response: We thank the reviewer for your appreciation.

Methods:

3. What were your indication to make a flap and which flap were used ? Specify the exclusion criteria.

Response: Free flap transfer for pharyngeal defect reconstruction after total laryngectomy would be performed if pharyngeal defect couldn’t primarily close without tension. The flap was most often harvested from the anterolateral thigh (n=160) followed by the anteromedial thigh (n=5) in our cohort. Patients who received partial laryngectomy or partial laryngopharyngectomy or patient didn’t receive total laryngectomy surgical procedure in our hospital would be excluded from our study. We have also updated the manuscript in the section of Method. (line 67 - 71 and line 153 - 156).

4. Line 80: you already define BMI in the abbreviation (line 70), just keep in this line BMI.

Response: We thank the reviewer for this insightful observation. We had change word from “Body mass index (BMI, kg/m^2^)” to “BMI”. (line 85)

Results:

5. line 114 = the cost is presented in Taiwan money and U.S. dollar. Please, present in the brackets the Euro value for European Physicians. Thus, you cover both U.S. and Europe.

Response: Thank you very much for this useful recommendation. We had added the information of Taiwan Dollar to Euro Exchange Rate in our revised manuscript. (line 122 - 123)

6. Briefly specify the RT protocol (number of Gy, fraction, etc.)

Response: We thank the reviewer for this suggestion. The radiation technique for these patients was intensity-modulated radiation therapy (IMRT). The primary radiation dose was between 6,600 and 7,000 cGy (2 cGy/fraction). We had added this description in the section of Result. (line 149 - 150)

Discussion:

7. Another biological point that is important to consider is the thyroid status. In practice, we usually observed that hypothyroidism patients have wound healing disorder and longer hospital stay. The lack of consideration of this point is a limitation that has to be addressed.

Response: We thank the reviewer for this great point. In previous study, Rosko et al. described postoperative hypothyroidism independently predicts postoperative wound-healing complications including pharyngocutaneous fistula formation in patients who underwent salvage total larygectomy [1]. In our cohort, most patients lack of thyroid function status in laboratory exam before/after total laryngectomy. Only 10 patients (10/213=4.7%) had thyroid function status before total laryngectomy and all of them were in euthyroid status. In postoperative period, 41 patients (41/213=19.2%) had checked thyroid function status during the hospitalization. Of them, 20 patients were in euthyroid status, 15 patients were in clinical hypothyroidism (decreased serum free T4 level and elevated serum TSH level) and 6 patients were in subclinical hypothyroidism (elevated serum TSH level and normal serum free T4 level). All patients diagnosed with hypothyroidism had been immediately treated with thyroxine according to our guideline. We have performed additional subgroup analyses between different postoperative thyroid function status. The association between thyroid function status and our interested outcome, including postoperative hospital stay and inpatient medical cost was showed in Table A. The univariate analysis didn’t show significance. The reason may be (1) the small number (n=41) in this analysis and (2) temporary low thyroid functional level postoperatively, because thyroxine supplement had been prescribed after hypothyroidism diagnosed. We have also updated the manuscript to reflect these findings. (line 253 – 263, 286 – 287)

Table A. Univariate analysis of postoperative thyroid function level impacting Postoperative hospital stay and Total inpatient medical cost (n=41)

| Variable | | Number | Postoperative hospital stay(≧18 days) | *p* | Total inpatient medical cost (≧ 379,000 NTD) | *p* |
| --- | --- | --- | --- | --- | --- | --- |
| Postoperative thyroid function level | Euthyroid status | 20 | 12 | 0.523 | 9 | 0.356 |
|  | Subclinical hypothyroidism | 6 | 2 |  | 2 |  |
|  | Clinical hypothyroidism | 15 | 9 |  | 10 |  |

NTD: New Taiwan dollars; 1 U.S. dollar = 30.2 NTD; 1 Euro is roughly equal to 34.0 NTD (according to the Bank of Taiwan, as of June 3, 2020)

8. Develop the limitation paragraph, heterogeneity between patients (indication/tumor localization, RT) etc. that may consist of heterogeneity in the study. The inclusion of non-SCC (papillary thyroid) is another bias.

Response: We thank the reviewer for this suggestion. We had added the limitation paragraph about this study in section of discussion (line 283 – 287)

Conclusion:

9. Remove “in total”. The sentence could be: Previous RT history and postoperative lower albumin level significantly impact the length of hospital stay.

Response: We thank the reviewer for this suggestion. We had changed this sentence in section of conclusion to “Previous RT history and postoperative lower albumin level significantly impact the length of hospital stay.” (line 290)

Reference:

1.	Rosko AJ, Birkeland AC, Bellile Eet al. Hypothyroidism and Wound Healing After Salvage Laryngectomy. Ann Surg Oncol 2018; 25:1288-1295.
